# Multicopper Oxidase-3 Is a Laccase Associated with the Peritrophic Matrix of *Anopheles gambiae*

**DOI:** 10.1371/journal.pone.0033985

**Published:** 2012-03-27

**Authors:** Minglin Lang, Michael R. Kanost, Maureen J. Gorman

**Affiliations:** 1 Department of Biochemistry, Kansas State University, Manhattan, Kansas, United States of America; 2 College of Life Science, Agricultural University of Hebei, Baoding, China; Universidade Federal do Rio de Janeiro, Brazil

## Abstract

The multicopper oxidase (MCO) family of enzymes includes laccases, which oxidize a broad range of substrates including polyphenols and phenylendiamines; ferroxidases, which oxidize ferrous iron; and several other oxidases with specific substrates such as ascorbate, bilirubin or copper. The genome of *Anopheles gambiae*, a species of mosquito, encodes five putative multicopper oxidases. Of these five, only AgMCO2 has known enzymatic and physiological functions: it is a highly conserved laccase that functions in cuticle pigmentation and tanning by oxidizing dopamine and dopamine derivatives. AgMCO3 is a mosquito-specific gene that is expressed predominantly in adult midguts and Malpighian tubules. To determine its enzymatic function, we purified recombinant AgMCO3 and analyzed its activity. AgMCO3 oxidized hydroquinone (a *p*-diphenol), the five *o*-diphenols tested, 2,2′-azino-bis(3-ethylbenzthiazoline-6-sulphonic acid) (ABTS), and *p*-phenylenediamine, but not ferrous iron. The catalytic efficiencies of AgMCO3 were similar to those of cuticular laccases (MCO2 orthologs), except that AgMCO3 oxidized all of the phenolic substrates with similar efficiencies whereas the MCO2 isoforms were less efficient at oxidizing catechol or dopa. These results demonstrate that AgMCO3 can be classified as a laccase and suggest that AgMCO3 has a somewhat broader substrate specificity than MCO2 orthologs. In addition, we observed AgMCO3 immunoreactivity in the peritrophic matrix, which functions as a selective barrier between the blood meal and midgut epithelial cells, protecting the midgut from mechanical damage, pathogens, and toxic molecules. We propose that AgMCO3 may oxidize toxic molecules in the blood meal leading to detoxification or to cross-linking of the molecules to the peritrophic matrix, thus targeting them for excretion.

## Introduction

The multicopper oxidase (MCO) family of enzymes includes laccases, which oxidize a broad range of substrates including polyphenols, aminophenols and phenylendiamines; ferroxidases, which oxidize ferrous iron; and several other oxidases with specific substrates such as ascorbate, bilirubin or copper [1]. Most studies of multicopper oxidases have focused on enzymes from fungi; however, multicopper oxidases have been found in a wide range of organisms including bacteria, yeast, plants, vertebrates and insects. All insect genomes analyzed to date contain at least two putative multicopper oxidase genes, designated MCO1 and MCO2 [2]. MCO2 orthologs are laccases that oxidize diphenols such as dopamine, *N*-acetyldopamine (NADA), and *N*-β-alanyldopamine (NBAD) during pigmentation and sclerotization of newly synthesized cuticle [3]. The substrate specificity and physiological functions of MCO1 orthologs and other insect MCOs have not been established, although there is evidence that a termite laccase, RfLac, functions in lignin degradation [4]. Other proposed but unconfirmed functions of insect multicopper oxidases are sclerotization of the egg case (in cockroaches), detoxification of monolignols (in leafhoppers), suppression of host immune responses (by wasp venom), defense against parasites (in mosquitoes), and iron metabolism (in fruit flies) [5]–[9].

Despite their diverse functions, multicopper oxidases are structurally similar, and most bind four copper atoms within two highly conserved copper centers [10]. A typical multicopper oxidase consists of three cupredoxin-like domains. Highly conserved residues in domains I and III coordinate the four copper ions, and residues in domains II and III form the substrate binding pocket [11]–[14]. The substrate specificity of multicopper oxidases is influenced by the size and shape of the substrate binding pocket, specific residues in the substrate binding pocket, and the difference in redox potential between the T1 copper and the substrate [11], [15]–[20]. Ferroxidases have conserved acidic residues that bind to ferrous iron [18], but conserved residues in the substrate binding pocket of laccases have not been identified.

The genome of *Anopheles gambiae*, a species of mosquito that transmits malaria parasites, encodes five putative MCO genes: MCO1, MCO2, and the mosquito-specific MCO3, 4 and 5 [2]. Other than their expression profiles in *A. gambiae*, not much is known about the mosquito-specific enzymes. AgMCO3 has little or no expression in the egg and larval stages, but its mRNA is abundant in the pupal and adult stages [2]. Studies of adult expression patterns in adult females demonstrated that AgMCO3 is expressed primarily in the midgut and Malpighian tubules, and AgMCO3 transcript abundance increases in these two tissues in response to a blood meal [2], [21]. These data strongly suggest that the main functions of AgMCO3 are associated with the midgut and Malpighian tubules. Note that AgMCO3 is not orthologous to the similarly named gene in *Drosophila melanogaster*; DmMCO3 (DmCG5959) has an unknown substrate preference and unknown expression patterns, and it may have a role in iron metabolism [2], [9].

The goal of this study was to determine the enzymatic function of AgMCO3 and to determine the location of AgMCO3 within the mosquito. To achieve these goals, we purified recombinant AgMCO3 from cultured insect cells, determined its pH optima, and analyzed its ability to oxidize several possible substrates. We found that the activity of AgMCO3 is similar to that of MCO2 orthologs, which are known to function as laccases. In addition, using immunohistochemistry, we determined that the likely location of AgMCO3 within the midgut is the peritrophic matrix.

## Results

### Sequence analysis

**Figure 1:**
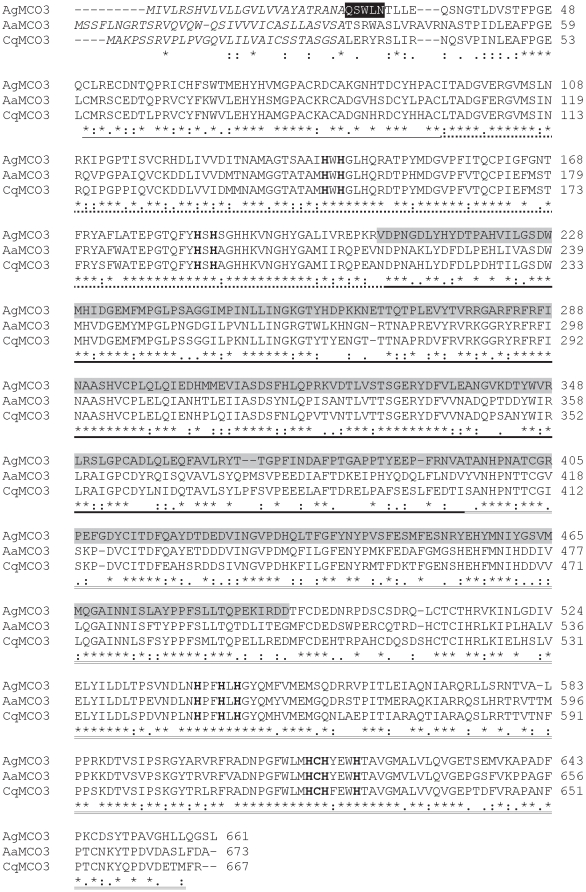
Alignment of amino acid sequences of MCO3 from *A. gambiae*, *A. aegpti*, and *C. quinquefaciatus*. Predicted signal peptides are in italicized text. The amino-terminal sequence of purified recombinant MCO3 is highlighted in black. A conserved cysteine-rich region is underlined. The three cupredoxin-like domains are indicated by dashed underlining (I), bold underlining (II), and double underlining (III). The 10 histidines and 1 cysteine that are predicted to bind copper are in bold text. The region of MCO3 that was used to generate polyclonal antiserum is highlighted in gray.

MCO3 orthologs from three species of mosquitoes (*A. gambiae*, *Aedes aegypti*, and *Culex quinquefaciatus*) share 60–76 percent amino acid sequence identity (Figure 1). Each MCO3 sequence contains a predicted signal peptide, suggesting that MCO3 orthologs are secreted proteins. In addition, the amino acid sequences contain three predicted cupredoxin-like domains and eleven predicted copper binding residues; these features suggest that MCO3 orthologs have oxidase activity. Predicting the substrate specificity of multicopper oxidases based on sequence information is often not possible; however, many ferroxidases have conserved acidic residues that bind to iron [18]. To identify potential iron binding residues in the MCO3 sequences, we aligned MCO3 and Fet3p sequences and searched for acidic residues in MCO3 that might correspond to the iron binding residues of Fet3p. We did not identify corresponding acidic residues in the MCO3 orthologs; however, the fairly low sequence similarity between the MCO3 orthologs and Fet3p resulted in gaps in the alignment that could have obscured positive identifications (Figure S1).

### Purification of AgMCO3

**Figure 2:**
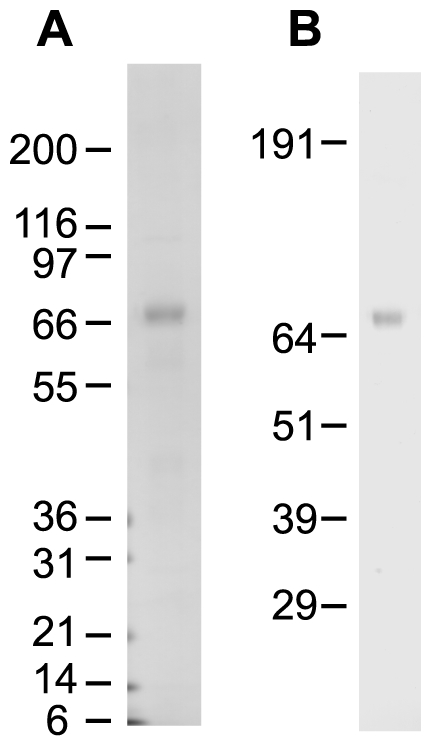
SDS-PAGE and immunoblot analysis of purified AgMCO3. A) Coomassie staining was used to detect 340 ng of purified enzyme. B) The identity of the purified protein was verified by immunoblot analysis.

**Figure 3:**
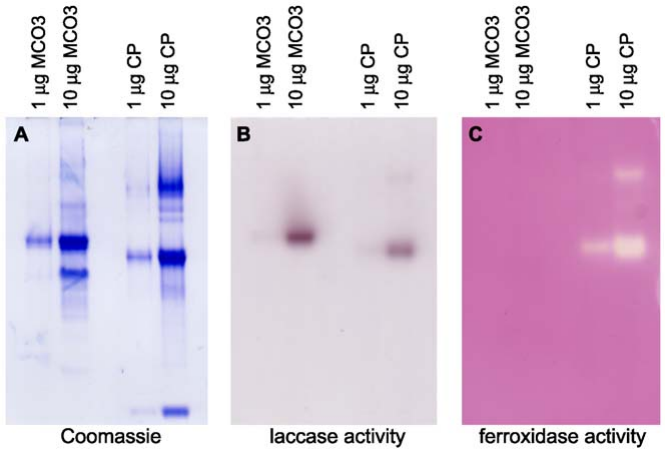
In-gel assay of ferroxidase activity. Triplicate native gels were run with 1 and 10 µg AgMCO3 and human ceruloplasmin. A) Coomassie staining. B) Assay for laccase activity using 9.2 mM *p*-phenylenediamine as the substrate. C) Assay for ferroxidase using 0.2 mM ferrous ammonium sulfate as the substrate. Note that both AgMCO3 and ceruloplasmin had detectable laccase activity (B), but only ceruloplasmin had detectable ferroxidase activity (C).

To allow us to directly investigate the substrate specificity of MCO3, we expressed recombinant AgMCO3 in cultured insect cells and then purified the secreted enzyme from the serum-fee medium with the use of concanavalin-A affinity chromatography followed by anion exchange chromatography. We purified approximately 1.5 mg AgMCO3 per liter of cell culture. The identity of AgMCO3 was verified by immunoblot analysis (Figure 2) and amino-terminal sequencing (Figure 1). SDS-PAGE followed by Coomassie staining demonstrated that recombinant AgMCO3 was fairly pure (Figures 2 and 3). The major contaminating band, visible when 10 µg of AgMCO3 was analyzed by native PAGE, did not visibly oxidize the laccase substrate *p*-phenylenediamine (Figure 3). In addition, we demonstrated previously that proteins that co-purify with other recombinant MCOs during the same lectin affinity and anion exchange chromatography procedures do not have detectable laccase activity when dopamine, hydroquinone or ABTS are used as the substrate [22]. The estimated molecular mass of recombinant AgMCO3 was approximately 73 kDa, which is consistent with a predicted mass of 71.8 kDa. The slightly higher apparent mass is likely the result of N-linked glycosylation (which is predicted at Asn12 and Asn60 of the mature protein, and is consistent with the binding of AgMCO3 to concanavalin-A).

### pH optima of AgMCO3

The optimal pH range for AgMCO3 activity was determined using several laccase substrates. pH values between 3.0–8.0 were tested. (Higher pH conditions were excluded from the analysis because they caused considerable autoxidation of most substrates.) We found that the optimal pH range of AgMCO3 for oxidation of phenolic substrates was 6.5–8.0 (Table 1 and Figure S2).

**Table 1:** pH optima for MCO3.

Substrate	pH optimum^a^
hydroquinone	6.5–7.0
catechol	7.0
dopa	7.5
dopamine	7.5–8.0
NADA	7.5–8.0
NBAD	7.0
ABTS	6.0

a The pH range tested was 3.0–8.0 in 0.5 unit increments.

### Laccase activity of AgMCO3

To determine whether AgMCO3 can be characterized as a laccase, we determined kinetic constants for AgMCO3 using a set of laccase substrates that included a *p*-diphenol (hydroquinone); five *o*-diphenols, including four that have been detected in extracts of *A. gambiae* (dopa, dopamine, NADA and NBAD) [22]; and one non-phenolic substrate (2,2′-azino-bis(3-ethylbenzthiazoline-6-sulphonic acid) (ABTS)). Activity was assayed by a spectrophotometric method that detected product formation. Kinetic curves were made by plotting the activity of AgMCO3 versus substrate concentration, the data were fit to the Michaelis-Menten equation by non-linear regression, and kinetic constants were estimated (Table 2 and Figure S3). The catalytic efficiencies (*k_cat_*/*K_m_*) associated with the phenolic substrates were similar (ranging from 66–323 min^−1^ mM^−1^). The catalytic efficiency associated with ABTS was low (18 min^−1^ mM^−1^), mainly because of a high *K_m_*, suggesting low affinity of AgMCO3 for ABTS. The catalytic efficiencies of AgMCO3 were comparable to those of MCO2 orthologs, except that AgMCO3 oxidized all of the phenolic substrates with similar efficiencies whereas MCO2 isoforms were less efficient at oxidizing catechol or dopa (Table 3).

**Table 2:** Kinetic constants for MCO3.

Substrate	pH	*k_cat_* (min^−1^)	*K_m_* (mM)	*k_cat_*/*K_m_* (min^−1^ mM^−1^)
hydroquinone	6.5	1571	5.2	302
catechol	7.0	306	2.1	146
dopa	7.5	93	1.4	66
dopamine	7.5	274	1.6	171
NADA	7.5	155	1.0	155
NBAD	7.0	711	2.2	323
ABTS	6.0	156	8.5	18

**Table 3:** Catalytic efficiencies (in min^−1^ mM^−1^) of AgMCO3 and four cuticular lacccases.

Substrate	AgMCO3	AgMCO2A^a^	AgMCO2B^a^	TcLac2A^a^	TcLac2B^a^
hydroquinone	302	114	315	213	276
catechol	146	29	45	40	54
dopa	66	30	8	10	11
dopamine	171	96	210	51	225
NADA	155	307	181	210	550
NBAD	323	219	190	165	282
ABTS	18	26	38	18	7

a The catalytic efficiencies of AgMCO2 and TcLac2 isoforms were reported previously [22].

### Lack of ferroxidase activity of AgMCO3

To determine whether AgMCO3 has ferroxidase activity, we used an in-gel ferroxidase assay. Recombinant AgMCO3 and human ceruloplasmin (a ferroxidase) were subjected to native PAGE in triplicate. One gel was stained with Coomassie blue to show the location and relative concentration of enzymes in the gels (Figure 3A). A second gel was incubated with *p*-phenylenediamine, a laccase substrate that is known to be oxidized by ceruloplasmin. This in-gel assay demonstrated that AgMCO3 and ceruloplasmin were active in the native gels (Figure 3B). The third gel was incubated with ferrous ammonium sulfate and then ferrozine (which reacts with ferrous iron to produce a purple pigment), and activity was detected as a clear band within a purple background. Using this assay, 1 µg of ceruplasmin had detectable activity, but 10 µg AgMCO3 had no detectable ferroxidase activity. These results, combined with the apparent lack of conserved iron binding residues, suggest that AgMCO3 is not a ferroxidase.

### Localization of AgMCO3

**Figure 4:**
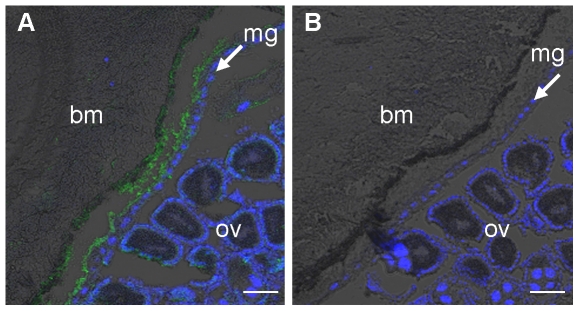
Localization of AgMCO3 in adult females. Cryosections of female mosquitoes (24 h post blood meal) were immunostained with AgMCO3 antiserum (A) or pre-immune serum (B). Anti-MCO3 antibodies were detected with Alexa Fluor 488 conjugated anti-rabbit IgG antibodies (green). Nuclei were stained with DAPI (blue). Immunoreactivity was detected between the blood meal and the midgut epithelial cells. This location is consistent with the hypothesis that AgMCO3 is present in the peritrophic matrix. Abbreviations: bm = blood meal, mg = midgut epithelial cells, ov = ovaries. Scale bar = 50 µm.

Previous analyses of AgMCO3 expression in adult female mosquitoes have shown that the mRNA is most abundant in the midgut and Malpighian tubules [2], [21]. Because AgMCO3 has a signal peptide, and recombinant AgMCO3 was secreted; we predicted that AgMCO3 would be synthesized by cells in the midgut and Malpighian tubules and then secreted into the lumen or hemolymph. To test this hypothesis, we used immunohistochemistry to determine the location of AgMCO3. In immunostained cryosections of females 24 hours post-blood meal, the strongest staining was of an acellular tissue between the midgut epithelial cells and the blood meal (Figure 4A–B). This result suggests that AgMCO3 is present in the peritrophic matrix, which is an extracellular matrix that is synthesized by the gut, secreted into the gut lumen, and surrounds the food bolus [23]. AgMCO3 was not detected in Malpighian tubules (data not shown), possibly because the sensitivity of our immunostaining technique was inadequate to detect a protein that may be secreted into the hemolymph or into the Malpighian tubule lumen and then excreted.

## Discussion

With the exception of the cuticular laccases (MCO2 orthologs), the enzymatic and physiological functions of insect multicopper oxidases have not been established. The main goal of this study was to determine the enzymatic function of AgMCO3. We found that the optimal pH range of AgMCO3 for oxidizing phenolic substrates was 6.5–8.0. This range is higher than that of insect MCO2 orthologs, which tend to exhibit maximum activity at pH 4.5–7.0 [3], [22]. In addition, the pH range is consistent with the slightly alkaline pH of the adult *A. gambiae* midgut [24]. AgMCO3 oxidized hydroquinone (a *p*-diphenol), the five *o*-diphenols tested, ABTS, and *p*-phenylenediamine but not ferrous iron. The catalytic efficiencies of AgMCO3 were similar to those of previously studied insect cuticular laccases, except that AgMCO3 oxidized all of the phenolic substrates with similar efficiencies whereas the MCO2 isoforms did not oxidize catechol or dopa very well. These results demonstrate that AgMCO3 can be classified as a laccase and suggest that AgMCO3 has a somewhat broader substrate specificity than MCO2 orthologs.

The second goal of this study was to determine the location of AgMCO3 within the adult female mosquito. In cryosections of females 24 hours post-blood meal, the only consistent immunostaining was of an acellular tissue between the midgut epithelial cells and the blood meal. This observation strongly suggests that AgMCO3 is associated with the peritrophic matrix. Corroborating this hypothesis is the previous identification of AgMCO3 as a putative peritrophic matrix protein by a proteomics approach (supplementary data in [25]). In addition, the low expression of AgMCO3 in the midguts of adult males [21] is consistent with the fact that adult males do not produce a peritrophic matrix. Based on all the available data, we conclude that AgMCO3 is a peritrophic matrix protein.

Based on our kinetic analysis of AgMCO3, we conclude that AgMCO3 functions as a laccase; however, we still do not know its physiological function. The peritrophic matrix is an extracellular matrix made up of chitin, proteins and glycoproteins [23]. It acts as a selective barrier between the food bolus and midgut epithelial cells, and it protects the epithelial cells from mechanical damage, some pathogens, and some toxic molecules, for example, heme, tannins, bacterial toxins and xenobiotics [23], [26]–[30]. In adult mosquitoes, it is synthesized in response to a blood meal and is excreted after the blood meal is digested; it is thought to protect the mosquito from the toxic effects of free heme and some xenobiotics by sequestering these molecules, which are excreted with the peritrophic matrix after blood digestion is complete [23], [26], [29], [30]. AgMCO3 may catalyze the cross-linking of peritrophic matrix proteins, but the type of cross-linking associated with insect laccases (quinone tanning and β-sclerotization [31]), are not known to occur in this tissue. A more likely hypothesis is that AgMCO3 oxidizes potentially toxic molecules in the blood meal, which might lead to detoxification or to cross-linking of the molecules to the peritrophic matrix, thus targeting them for excretion.

## Materials and Methods

### Mosquito culture

The G3 strain of *Anopheles gambiae* was obtained from the Malaria Research and Reference Reagent Resource Center. The mosquitoes were reared as described previously [32] with minor modifications. Briefly, larvae were reared at 27°C and were fed a mixture of baker's yeast and ground fish food. Adults were maintained at 27°C with 85% relative humidity and were fed 10% sucrose. Adult females were fed heparinized equine blood with a Hemotek membrane feeder.

### Sequence analyses

Clustal W [33] was used to align the predicted amino acid sequences of MCO3 from *A. gambiae* (GenBank ID: EF592176.2, with one amino acid difference as described below), *A. aegypti* (GenBank ID: XP_001653727.1), and *C. quinquefaciatus* (GenBank ID: XP_001842487.1). Signal sequences were predicted by Signal P [34]. Insect MCO-specific cysteine rich regions were defined as previously described [35]. Boundaries of the putative cupredoxin-like domains were estimated by aligning MCO3 sequences with the sequence of a fungal laccase, *Trametes versicolor* laccaseIIIb (TvLacIIIb, PDB ID: 1KYA), which has a solved crystal structure, and using SCOP [36] to define the boundaries of the cupredoxin-like domains of TvLacIIIb. Clustal W [33] was also used to align the predicted amino acid sequences of the three cupredoxin-like domains of the MCO3 sequences described above and the yeast Fet3p sequence (GenBank ID: NP_013774.1). N-linked glycosylation was predicted by NetNGlyc (www.cbs.dtu.dk/services/NetNGlyc).

### Recombinant protein expression

The AgMCO3 cDNA sequence deposited in GenBank (GenBank ID: EF592176.2) had a probable error in the predicted signal peptide; therefore, for this study, we used a cDNA (obtained by RT-PCR from adult female RNA) that was identical to the GenBank sequence except that the codon for residue 15 encoded a leucine instead of a proline. Recombinant AgMCO3 was expressed using the Bac-to-Bac baculovirus expression system (Invitrogen). The AgMCO3 cDNA was cloned into pFastBac1, the DNA sequence was verified to be correct, and a recombinant baculovirus was generated. Plaque assays were used to determine titers of amplified virus stocks.

For expression, 1.6–2 liters of Sf9 cells (at 2×10^6^ cells/ml in Sf-900 II serum free medium supplemented with 0.1 mM copper sulfate) were infected with baculovirus at a multiplicity of infection of 2, and cells were incubated at 28°C with shaking for 48 hours. Cells were removed by centrifugation at 500×*g* for 10 min. To reduce degradation of AgMCO3, two protease inhibitors, 10 µM E64 and 0.5 mM *p*-aminobenzamidine, were added to the conditioned medium. Glycosylated proteins in the cell culture medium were bound to concanavalin-A-Sepharose and eluted with 0.5 M methyl-α-D-mannopyranoside in 20 mM Tris-HCl, 0.5 M NaCl, 0.5 mM *p*-aminobenzamidine, pH 7.4. Eluted proteins were dialyzed twice against 20 mM Tris-HCl, 0.5 mM *p*-aminobenzamidine, pH 8.0 or 8.2. Dialyzed proteins were bound to a Q-Sepharose column and eluted with a linear gradient (0–1 M) of NaCl in 20 mM Tris, pH 8.0. Fractions containing a high concentration of AgMCO3 and a low concentration of contaminating proteins were pooled. Purified AgMCO3 was stored at −20°C in the presence of 50% glycerol or was concentrated to approximately 3 µg/µl with the use of Amicon Ultracel 10k centrifugation units before storing small aliquots at −80°C. The yield of recombinant AgMCO3 was 1–2 mg per liter of cell culture.

### Estimation of enzyme concentration

The concentration of purified recombinant AgMCO3 was estimated by performing SDS-PAGE analysis of two dilutions of AgMCO3 and several dilutions of bovine serum albumin (BSA). The gels were stained with Coomassie blue, and the band intensities of the recombinant proteins and the BSA standards were compared.

### Amino-terminal sequencing

Purified recombinant AgMCO3 was subjected to SDS-PAGE and transferred to a PVDF membrane. Proteins were lightly stained with Coomassie R, and the protein band was excised from the membrane. To de-block the amino-terminus, the membrane containing AgMCO3 was incubated in 200 µl 0.5% polyvinylpyrolidone in 0.1 M acetic acid for 30 min at 37°C, rinsed with water, submerged in 2 mU *Pfu* pyroglutamate aminopeptidase (TaKaRa) in 100 µl of 50 mM sodium phosphate, 10 mM DTT, 1 mM EDTA, pH 7.0, flushed with nitrogen gas, and incubated for 5 h at 50°C. Edman protein sequencing was done by Dr. Kathleen Schegg at the Nevada Proteomics Center. An ABI 492 Procise sequencer was used to determine the first five residues of AgMCO3.

### Production of polyclonal antiserum and immunoblot analysis

Polyclonal antiserum was generated against residues V207 - D492 of AgMCO3 (see Figure 1). This portion of AgMCO3 corresponds to one of the less conserved regions of insect multicopper oxidases. The corresponding partial cDNA was amplified by PCR and cloned into an *E. coli* expression vector, pET32 (Novagen). The truncated fusion protein was expressed in the Origami B strain of *E. coli* (Novagen) and purified by nickel affinity chromatography under denaturing conditions. The purified protein was concentrated to approximately 2 mg/ml with the use of an Amicon Centriplus YM-10 centrifugal filter device, and 0.5 mg was subjected to SDS-PAGE. The gel was lightly stained with 0.05% Coomassie R in water, and the protein band was excised and sent to Cocalico Biologicals Incorporated (Reamstown, PA) for the production of polyclonal antiserum in a rabbit.

The sensitivity and specificity of AgMCO3 antiserum (KSU181) was assessed by immunoblot analysis. Five and 50 ng of the AgMCO3 antigen (V207 - D492) and the corresponding regions of the other *A. gambiae* multicopper oxidases (MCO1, MCO2B, MCO4 and MCO5 [2]) were subjected to reducing SDS-PAGE followed by protein transfer to nitrocellulose and immunodetection using using a 1∶2,000 dilution of the polyclonal antiserum generated against MCO3. This method detected 5 ng and 50 ng of AgMCO3 antigen, but no bands corresponding to the other MCOs were visible (data not shown).

### Laccase activity assays

The laccase substrates used were *N*-acetyldopamine (NADA), 2,2′-azino-bis(3-ethylbenzthiazoline-6-sulphonic acid) diammonium salt (ABTS), catechol, L-dopa, dopamine hydrochloride and hydroquinone (all purchased from Sigma-Aldrich), and *N*-β-alanyldopamine hydrochloride (NBAD), which was provided by the National Institute of Mental Health's Chemical Synthesis and Drug Supply Program. The molar extinction coefficients (in M^−1^ cm^−1^) of the products of interest were: dopaminochrome, ε_475_ = 3,058 [37]; NADA quinone and NBAD quinone, ε_390_ = 1,100 [38]; dopachrome, ε_475_ = 3,600 [38]; *o*-benzoquinone, ε_450_ = 2,211 [39]; *p*-benzoquinone ε_248_ = 17,252 [39]; and ABTS cation, ε_414_ = 36,000 [39].

Laccase activity assays were performed as described previously with minor modifications [22]. Reactions to determine laccase activity were made by mixing 0.5 µg AgMCO3 with substrate in a total volume of 200 µl and detecting product formation with a microplate spectrophotometer by observing the change in absorbance over time. To account for autoxidation of substrates, reactions with no enzyme were included, and the slopes of these “blank” reactions were subtracted from the slopes of the enzyme-containing reactions. The reactions used to determine the pH optima of AgMCO3 contained 0.5 µg enzyme and 2 mM substrate in citrate-phosphate buffer, pH 3.0–8.0. The reactions used to determine the kinetic properties of AgMCO3 contained 0.5 µg enzyme and a range of substrate concentrations, and the choice of buffer was dependent on the optimum pH for each substrate. Reactions at pH 6.0–6.5 were buffered by 0.1 M Mes, and reactions at 7.0–7.5 were buffered by 0.1 M sodium phosphate. All assays were done in triplicate. Kinetic curves were made by plotting the activity of AgMCO3 versus substrate concentration. The data were fit to the Michaelis-Menten equation by non-linear regression using GraphPad Prism software. The kinetic constants *V_max_* and *K_m_* were estimated from thefitted data.

### In-gel activity assays

Recombinant AgMCO3 and human ceruloplasmin (Sigma) were subjected to native PAGE using 8% polyacrylamide gels and Tris glycine buffer. Gels were run in triplicate. One gel was stained with Coomassie R to show the location and relative concentration of enzymes in the gels. The second gel was equilibrated in 0.1 M sodium acetate, pH 6.0, for 15 minutes, and then incubated in 9.2 mM *p*-phenylenediamine (Sigma) for 2.5 h to detect laccase activity. The third gel was used to detect ferroxidase activity as desribed previously [40] with minor modifications. Briefly, the gel was equilibrated in 0.1 M sodium acetate, pH 6.0, for 15 minutes, incubated in 0.2 mM ferrous ammonium sulfate in 0.1 M sodium acetate, pH 6.0, for 2 h at 37°C to allow enzymes to oxidize ferrous iron to ferric iron, and then incubated briefly in 15 mM FerroZine (Acros Organics), which reacts with ferrous iron to produce a purple pigment. Ferroxidase activity results in a clear band (corresponding to a low concentration of ferrous iron) surrounded by a purple background.

### Immunohistochemistry

To detect AgMCO3 in the mosquito, immunostaining of cryosections was performed. Four day old female mosquitoes were given a blood meal and incubated at 27°C for 24 hours. Prior to fixation, the legs and wings were removed, and a small tear was introduced in the thoracic cuticle to allow fixative to penetrate. The mosquitoes were fixed in 4% paraformaldehyde in phosphate buffered saline (PBS) at 4°C overnight. The fixed mosquitoes were infiltrated with increasing concentrations of sucrose (12, 15, 18 and 20% in PBS) at 4°C. The samples were placed in a drop of OCT compound (Tissue Tek) on a small piece of filter paper and then frozen by dropping them into a tube of 2-methylbutane that was cooled with liquid nitrogen. Longitudinal sections (12 µm) were made with a Leica 3050 cryostat. Cryosections were rinsed three times with PBST (PBS with 0.1% Tween 20). The sections were permeabilized by incubating in 0.1% Triton X-100 in Tris buffered saline for 10–15 min. Sections were blocked for 1 h in PBST containing 2% bovine serum albumin. After blocking, sections were incubated for 2 h with AgMCO3 antiserum or pre-immune serum (diluted 1∶200 in blocking buffer), rinsed three times for 5 min with PBST, incubated for 1 h with Alexa Fluor 488 conjugated goat anti-rabbit IgG (Invitrogen, diluted 1∶500 in blocking buffer), and rinsed three times with PBST. Nuclei were stained by incubating the sections with 2 µg/ml 4′, 6-diamidino-2-phenylindole dihydrochloride (DAPI) for 10–15 min. The slides were rinsed three times with PBST, and sections were covered with FluorSave reagent (Calbiochem). Sections were analyzed with a Zeiss LSM 510 META laser scanning confocal microscope using excitation wavelengths of 405 and 488 nm and a 10× Plan-Neofluar objective with a 0.3 numerical aperture.

## Supporting Information

Figure S1 **Alignment of three mosquito MCO3 sequences and the yeast Fet3p sequence.** Residues highlighted in yellow are iron-binding residues. Non-alignable sequences at the amino- and carboxyl-termini were omited from the alignment.
(PDF)

Figure S2 **pH profiles of MCO3 activity.** Assays were performed with 2 mM substrate in citrate-phosphate buffer. Data are expressed as mean ± standard deviation (n = 3).
(PDF)

Figure S3 **Kinetics of MCO3 activity.** Data are expressed as mean ± standard deviation (n = 3). Non-linear regression was used to fit the data to the Michaelis-Menten equation (dotted lines).
(PDF)
