# Supplementary material for: Multicopper Oxidase-3 Is a Laccase Associated with the Peritrophic Matrix of Anopheles gambiae
Source: PLoS One. 2012 Mar 27;7(3):e33985. doi: 10.1371/journal.pone.0033985 (PMC3313952; doi:10.1371/journal.pone.0033985)
Supplement: Figure S1 — Alignment of three mosquito MCO3 sequences and the yeast Fet3p sequence. Residues highlighted in yellow are iron-binding residues. Non-alignable sequences at the amino- and carboxyl-termini were omited from the alignment. (PDF) [file pone.0033985.s001.pdf]

```
AgMCO3   RGVMSLNRKIPGPTISVCRHDLIVVDITNAMAGTSAAIHWHGLHQRATPYMDGVPFITQC 60
AaMCO3   RGVMSINRQVPGPAIQVCKDDLIVVDMTNAMGGTATAMHWHGLHQRDTPHMDGVPFVTQC 60
CqMCO3   RGVMSINRQIPGPPIQVCKDDLVVIDMMNAMGGTATAMHWHGLHQRDTPYMDGVPFVTQC 60
ScFet3   RPVITCNGQFPWPDITVNKGDRVQIYLTNGMNNTNTSMHFHGLFQNGTASMDGVPFLTQC 60
         * *:: * :.* * * * : * : : : *.* .* :::*:***.*. *. ******:***

AgMCO3   PIGFGNTFRYAFLAT-EPGTQFYHSHSGHHKVNGHYGALIVREPKRVDPNGDLYHYDTPA 119
AaMCO3   PIEFMSTFRYAFWAT-EPGTQFYHSHAGHHKVNGHYGAMIIRQPEVNDPNAKLYDFDLPE 119
CqMCO3   PIEFMSTFRYSFWAT-EPGTQFYHSHAGHHKVNGHYGAMIIRQPEANDPNAHLYDFDLPD 119
ScFet3   PIAPGSTMLYNFTVDYNVGTYWYHSHTDGQYEDGMKGLFIIKD------DSFPYDYDEEL 114
         **   .*: * * .  : ** :****:. :  :*  * :*:::      :.  *.:*

AgMCO3   HVILGSDWMRIDGEMFMPGLPSAGGIMPINLLINGKGTYHDPKKNETTQTPLEVYTVRRG 179
AaMCO3   HLIVASDWMHVDGEMYMPGLPNGDGILPVNLLINGRGTWLKHNGN-RTNAPREVYRVRKG 178
CqMCO3   HTILGSDWMHVDGEMFMPGLPSSGGILPKNLLINGKGTYTYENGT-TTNAPRDVFRVRKG 178
ScFet3   SLSLSEWYHDLVTDLTKSFMS----------VYNPTGAEPIPQNLIVNNTMNLTWEVQPD 164
            :.. :  :  ::  . :.          : *  *:    :    .::   .: *: .

AgMCO3   ARFRFRFINAASHVCPLQLQIEDHMMEVIASDSFHLQPRKVDTLVSTSGERYDFVLEANG 239
AaMCO3   GRYRFRFINAASHVCPLELQIANHTLEIIASDSYNLQPISANTLVTTSGERYDFVVNADQ 238
CqMCO3   GRYRFRFINAASHVCPLELQIENHPLQIIASDSFNLQPVTVNTLVTTSGERYDFVVNADQ 238
ScFet3   TTYLLRIVNVGGFVS-QYFWIEDHEMTVVEIDGITTEKNVTDMLYITVAQRYTVLVHTKN 223
           : :*::*....*.   : * :* : ::  *.   :   .: *  * .:** .::.:.

AgMCO3   VKDTYWVRLRSLGPCADLQLEQFAVLRYT--TGPFINDAFPTGAPPTYEEP-FRNVATAN 296
AaMCO3   PTDDYWIRLRAIGPCDYRQISQVAVLSYQPMSVPEEDIAFTDKEIPHYQDQLFLNDVYVN 298
CqMCO3   PSANYWIRLRAIGPCDYLNIDQTAVLSYLPFSVPEEELAFTDRELPAFSESLFEDTISAN 298
ScFet3   DTDKNFAIMQKFDDTMLDVIPSDLQLNATSYMVYNKTAALPTQNYVDSIDN--------- 274
          .   :  :: :.      : .   *            *:.        :

AgMCO3   HPNATCGRPEFGDYCITDFQAYDTDEDVINGVPDHQLTFGFYNYPVSFESMFESNRYEHY 356
AaMCO3   HPNTTCGVSKP-DVCITDFQAYETDDDVINGVPDMQFILGFENYPMKFEDAFGMGSHEHF 357
CqMCO3   HPNTTCGISKP-DVCITDFEAHSRDDSIVNGVPDHKFILGFENYRMTFDKTFGENSHEHF 357
ScFet3   --------------FLDDFYLQPYEKEAIYGEPDHVITV--------------------- 299
                        : **     :.. : * **  : .

AgMCO3   MNIYGSVMMQGAINNISLAYPPFSLLTQPEKIRDDTFCDEDNRPDSCSDRQ-LCTCTHRV 415
AaMCO3   MNIHDDIVLQGAINNISFTYPPFSLLTQTDLITEGMFCDEDSWPERCQTRD-HCTCIHRL 416
CqMCO3   MNIHDDVVLQGAINNLSFSYPPFSMLTQPELLREDMFCDEHTRPAHCDQSDSHCTCIHRL 417
ScFet3   -----DVVMDNLKNGVNYAFFNNITYTAP---KVPTLMTVLSSGDQANNSEIYGSNTHTF 351
              .::::.  *.:. ::      * .       :    .    ..   :   :  * .

AgMCO3   KINLGDIVELYILDLTPSVNDLNHPFHLHGYQMFVMEMSQDRRVPITLEIAQNIARQRLL 475
AaMCO3   KIPLHALVELYILDLTPEVNDLNHPFHLHGYQMYVMEMGQDRSTPITMERAQKIARRQSL 476
CqMCO3   KIELHSLVELYILDLSPDVNPLNHPFHLHGYQMHVMEMGQNLAEPITIARAQTIARAQSL 477
ScFet3   ILEKDEIVEIVLNNQDTGT----HPFHLHGHAFQTIQRDRTYDDALG-EVPHSFDPD--- 403
          :    :**: : :  . .    *******: : .:: .:    .:    .:.:

AgMCO3   SRNTVA-LPPRKDTVSIPSRGYARVRFRADNPGFWLMHCHYEWHTAVGMALVLQVGETSE 534
AaMCO3   HRTRVTTMPPKKDTVSVPSKGYTRVRFVADNPGFWLMHCHYEWHTAVGMVLVLQVGEPGS 536
CqMCO3   RRTTVTNFPPSKDTVSIPSKGYTRLRFRADNPGFWLMHCHFEWHTAVGMALVVQVGEPTD 537
ScFet3   NHPAFPEYPMRRDTLYVRPQSNFVIRFKADNPGVWFFHCHIEWHLLQGLGLVLVEDPFGI 463
          :  ..  *  :**: : .:.   :** *****.*::*** ***   *: **:  .

AgMCO3   MVKAPADFPKC-------------- 545
AaMCO3   FVKPPAGFPTCNKYTPDVDASLFDA 561
CqMCO3   FVRAPANFPTCNKYQPDVDETMFR- 561
ScFet3   QDAHSQQLSENHLEVC--------- 479
             .  :.
```
