# Supplementary figures and images for: Multicopper Oxidase-3 Is a Laccase Associated with the Peritrophic Matrix of Anopheles gambiae
Source: PLoS One. 2012 Mar 27;7(3):e33985. doi: 10.1371/journal.pone.0033985 (PMC3313952; doi:10.1371/journal.pone.0033985)

**Hydroquinone**

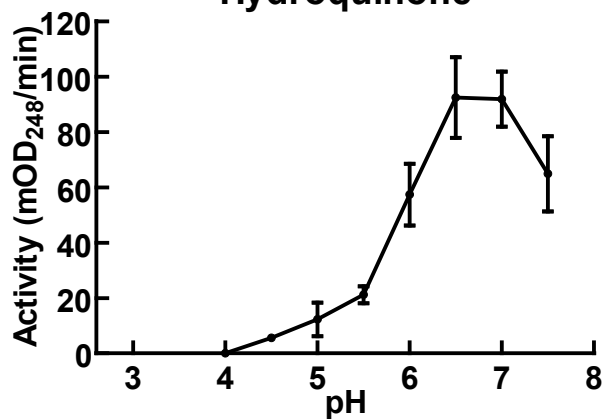

**Catechol**

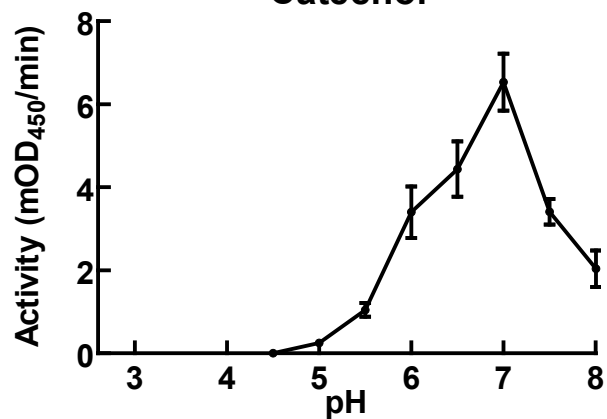

**Dopa**

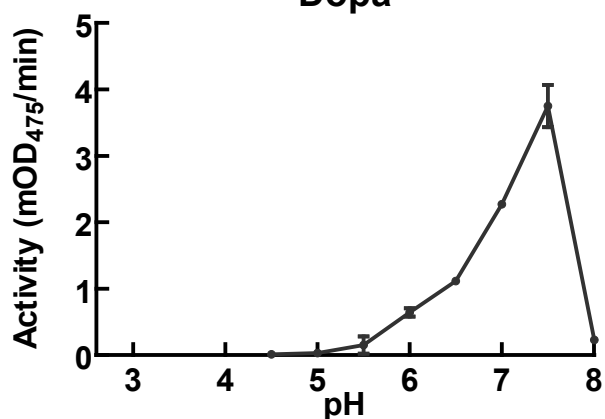

**Dopamine**

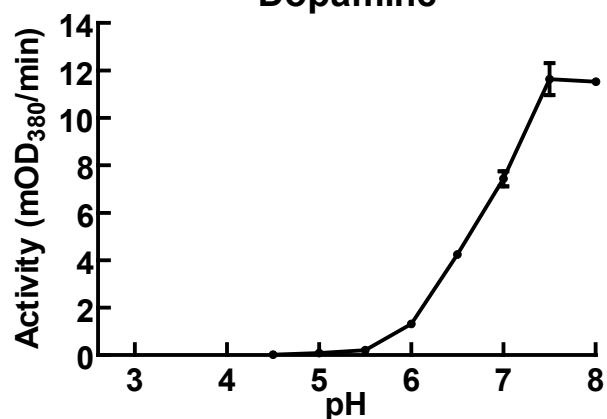

**NADA**

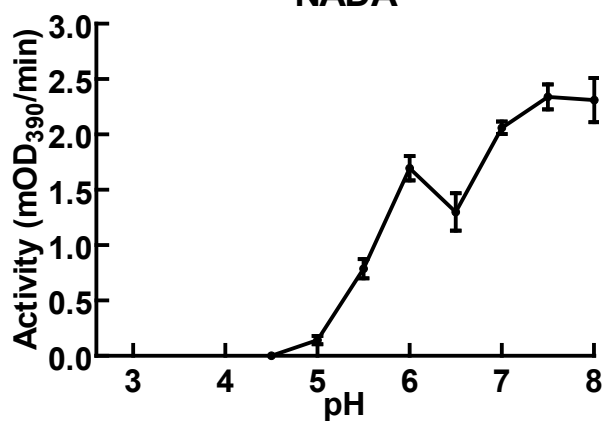

**NBAD**

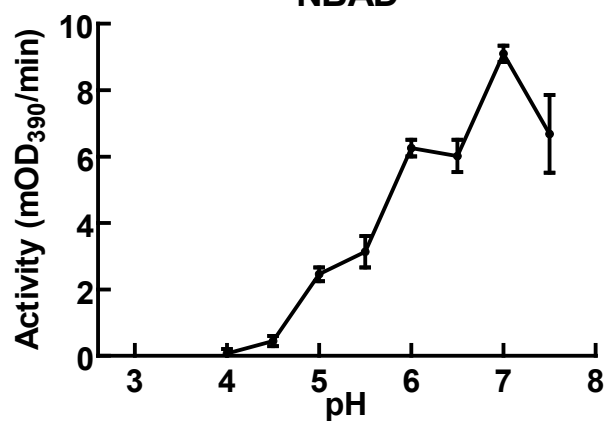

**ABTS**

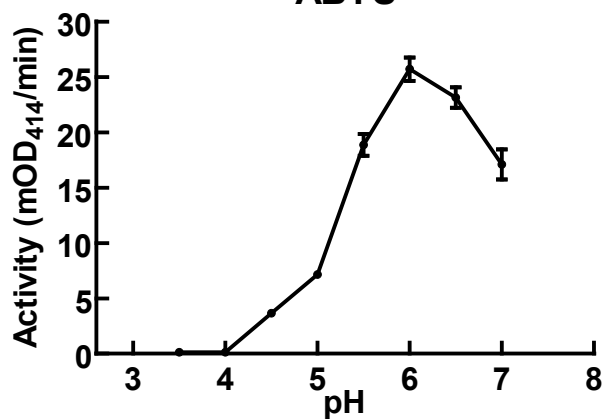

Supplement: Figure S2 — pH profiles of MCO3 activity. Assays were performed with 2 mM substrate in citrate-phosphate buffer. Data are expressed as mean ± standard deviation (n = 3). (PDF) [file pone.0033985.s002.pdf]

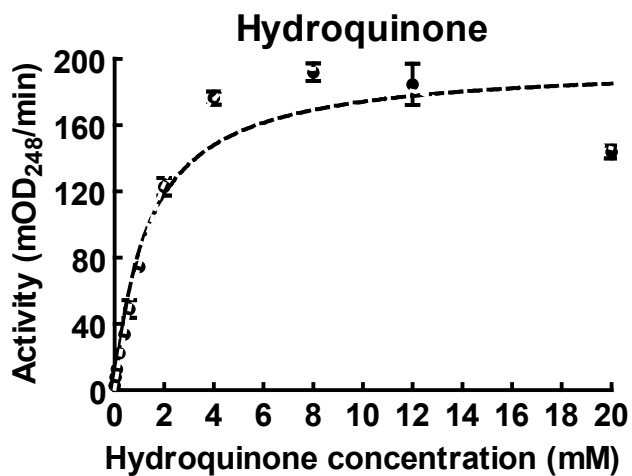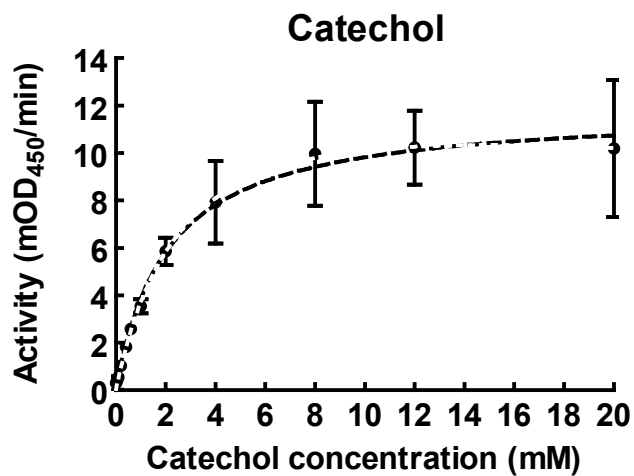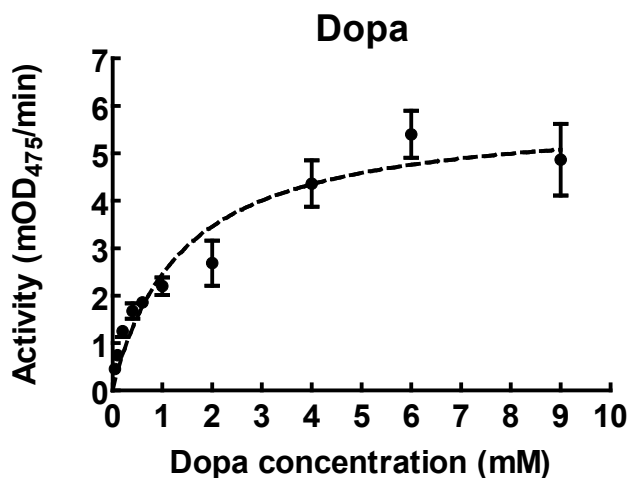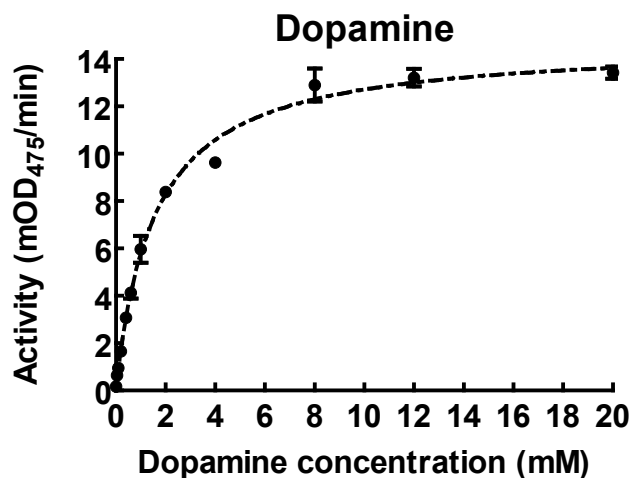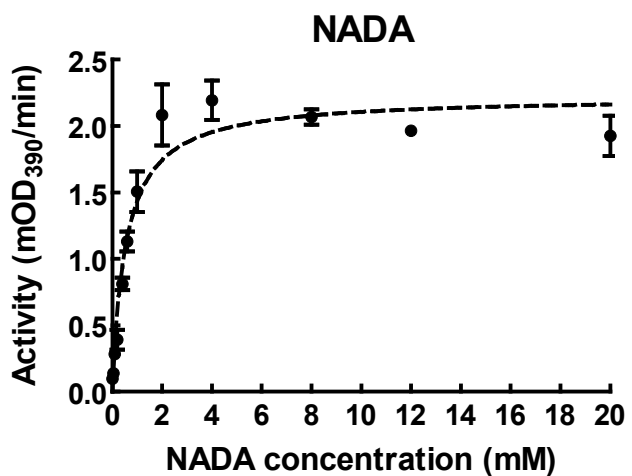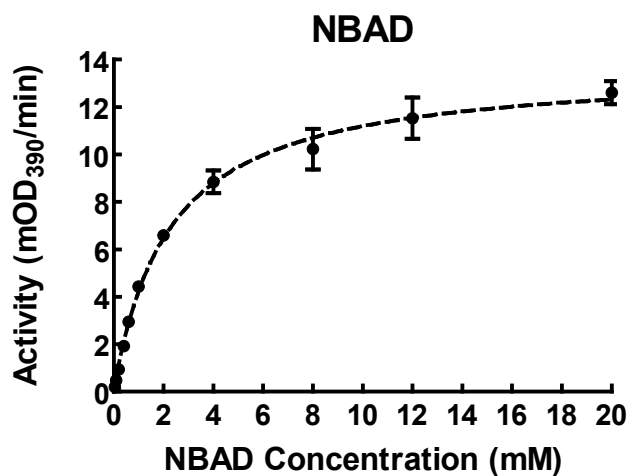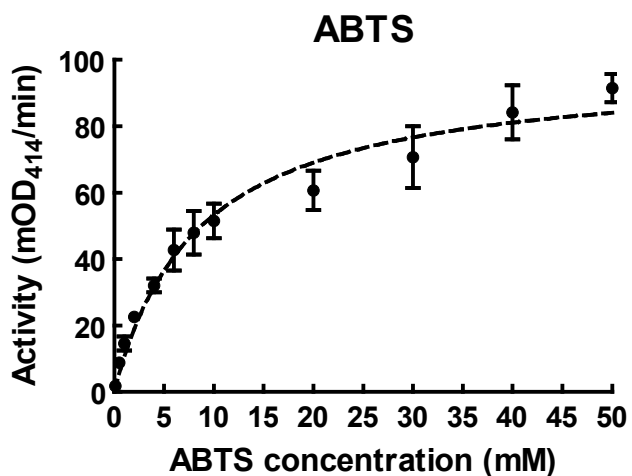

Supplement: Figure S3 — Kinetics of MCO3 activity. Data are expressed as mean ± standard deviation (n = 3). Non-linear regression was used to fit the data to the Michaelis-Menten equation (dotted lines). (PDF) [file pone.0033985.s003.pdf]
